# A Case of Pediatric Subcutaneous Panniculitis-like T-Cell Lymphoma Successfully Treated with Immunosuppressive Therapy

**DOI:** 10.3390/children12081029

**Published:** 2025-08-05

**Authors:** Min Chong Kim, Dong Hoon Shin, Jae Min Lee

**Affiliations:** 1 Department of Pathology, Yeungnam University Medical Center, Daegu 42415, Republic of Korea; mckimpath@yu.ac.kr; 2 Department of Dermatology, College of Medicine, Yeungnam University, Daegu 42415, Republic of Korea; dhshin@med.yu.ac.kr; 3 Department of Pediatrics, Pusan National University School of Medicine, Pusan National University Children’s Hospital, Yangsan 50612, Republic of Korea

**Keywords:** subcutaneous panniculitis-like T-cell lymphoma, immunosuppressive therapy, C9 gene mutation

## Abstract

**Highlights:**

**What are the main findings?**

- A 14-year-old female with SPTCL (without HLH) was successfully treated using immunosuppressive therapy alone (methylprednisolone and cyclosporine A), leading to rapid symptom resolution, the normalization of laboratory markers (e.g., ferritin, CRP, ESR), and sustained remission without relapse for over 1 year after tapering and discontinuation.
- A heterozygous C9 gene mutation (c.346C>T, p.Arg116Ter) was identified via next-generation sequencing in a pediatric SPTCL patient, suggesting a potential link between partial complement system dysfunction (impaired MAC formation) and immune dysregulation contributing to SPTCL pathogenesis.

**What is the implication of the main findings?**

- This case supports considering immunosuppressive therapy as an initial treatment option for pediatric SPTCL without HLH, highlighting its efficacy in achieving long-term remission and adding to the evidence that genetic factors like C9 mutations may play a role in disease presentation resembling autoimmune conditions.

**Abstract:**

**Introduction:** Subcutaneous panniculitis-like T-cell lymphoma (SPTCL) is a very rare subtype of cutaneous T-cell lymphoma. It is characterized by the neoplastic infiltration of subcutaneous adipose tissue. Its clinical presentation, including subcutaneous nodules, fever, and systemic symptoms, often mimics inflammatory panniculitis, making diagnosis difficult. **Case Presentation:** This case report describes a 14-year-old female presenting with fever, limb pain, swelling, and subcutaneous nodules, who was ultimately diagnosed with SPTCL via punch biopsy and BIOMED-2 clonality assays, confirming positive T-cell receptor-γ chain gene rearrangement. Positron emission tomography–computed tomography revealed diffuse subcutaneous involvement across multiple body regions. Methylprednisolone and cyclosporine A treatment rapidly resolved her symptoms, with laboratory parameters, including ferritin and inflammatory markers, showing significant improvement. Next-generation sequencing identified a heterozygous C9 gene mutation (c.346C>T, p.Arg116Ter), adding a novel genetic dimension to the case. Following a tapered discontinuation of immunosuppressive therapy, the patient achieved sustained remission without relapse for over 1 year. **Conclusions:** We report a case of adolescent SPTCL treated with immunosuppressive therapy and suggest that immunosuppressive therapy should be considered before chemotherapy in pediatric patients with SPTCL but without HLH.

## 1. Introduction

Subcutaneous panniculitis-like T-cell lymphoma (SPTCL) is a rare cutaneous T-cell lymphoma that affects subcutaneous adipose tissue, often mimicking inflammatory panniculitis [1,2]. It was first reported as a new T-cell lymphoma subtype by Gonzalez et al. in 1991 [3]. The 2005 World Health Organization–European Organisation for Research and Treatment of Cancer (WHO-EORTC) classification limited SPTCL to the α/β T-cell phenotype and differentiated the γ/δ phenotype as a separate disease; meanwhile, the 2016 update of the WHO-EORTC classification further refined the diagnostic criteria for SPTCL and clarified its histologic and molecular features [4,5]. SPTCL accounts for <1% of non-Hodgkin lymphomas, with a median diagnosis age of 36–40 years. However, cases occur across all ages, including children.

The presence of hemophagocytic lymphohistiocytosis (HLH) is a critical prognostic factor in SPTCL, which is observed in approximately 15–20% of patients with SPTCL and greatly affects outcomes [6]. In pediatric and younger patient populations, the incidence of HLH is high, often necessitating more aggressive therapeutic approaches [7,8]. Although HLH can reduce the 5-year overall survival (OS) rate by nearly half, prompt diagnosis and intensive intervention, including high-dose chemotherapy, have shown potential for achieving remission [6].

Currently, there is no standardized protocol for SPTCL treatment, which includes immunosuppressive therapies (e.g., steroids, low-dose methotrexate, cyclosporine A [CyA], and hydroxychloroquine) and chemotherapy regimens (e.g., CHOP). According to the Thai lymphoma study group, CyA-based immunosuppressive regimens may offer superior complete remission (CR) rates compared with chemotherapy, particularly for patients with limited skin involvement [9]. However, conflicting findings from other studies underscore SPTCL’s heterogeneity and the complexities in establishing universal treatment guidelines because of its rarity and diverse patient demographics.

Pediatric SPTCL represents a particularly challenging subset because of its infrequent occurrence, greatly hampering the conduct of prospective therapeutic trials [2,10]. Consequently, most data on pediatric SPTCL are derived from singular case reports. Despite this, pediatric patients appear to demonstrate a relatively superior response to chemotherapy compared to adults, potentially reflecting differences in tumor biology or immune environments between age groups [8,11,12,13]. Furthermore, recent studies have highlighted a high frequency of HAVCR2 gene mutations in pediatric patients with SPTCL [14,15]. These mutations are associated with an earlier age of onset and a high rate of autoantibody positivity. This suggests that SPTCL may present features resembling autoimmune diseases in these patients, emphasizing the need for genetic testing and careful differentiation during diagnosis.

Here, we present the case of an adolescent female patient diagnosed with SPTCL who achieved successful remission with immunosuppressive therapy. This case report contributes to the limited body of literature on pediatric SPTCL and further supports the consideration of immunosuppressive therapy as an initial treatment option in pediatric patients with SPTCL, especially in the absence of HLH.

## 2. Case Report

A 14-year-old female patient had intermittent fever and pain in both legs for 6 months prior to hospital admission. Three months ago, her legs became stiff, and she was hospitalized at a local medical center for cellulitis and received antibiotic treatment. Two months ago, she presented with symptoms such as fever (negative), limb swelling, and a positive pain test. By the end of May, she developed facial swelling, bruising under the eyes, palpable nodules on the arms and legs, and skin sensitivity to temperature. She experienced a fever more than once a day and was admitted to the hospital through the outpatient department.

**Figure 1:**
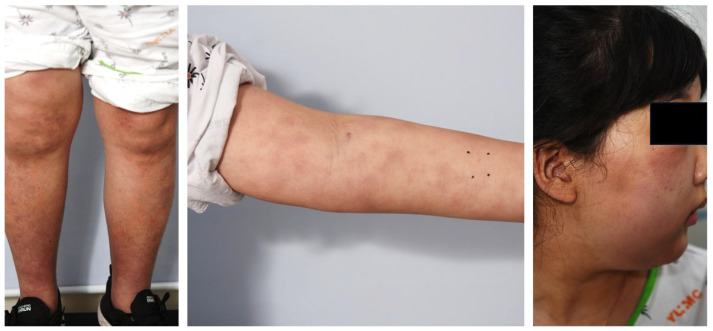
Clinical findings of the leg, arm, and face on the first visit.

Her past medical history revealed no significant findings. At the time of admission, her weight, body temperature, height, pulse rate, respiratory rate, and blood pressure were 57.5 kg, 39.1 °C, 157.6 cm, 119 bpm, 20 breaths per minute, and 150/100 mmHg, respectively. The patient appeared acutely ill. HEENT findings included anicteric sclera, non-pale conjunctiva, and intact tympanic membranes and throat. Chest examination revealed a regular heartbeat without murmurs and clear breathing sounds without rales. Her abdomen was soft and flat, with normal bowel sounds and no hepatosplenomegaly. Her face, trunk, and extremities showed generalized swelling, with multiple skin lesions (Figure 1).

Her initial complete blood count revealed a leukocyte count, hemoglobin, and platelet count of 4.58 × 10^9^/L, 12.1 g/dL, and 239 × 10^9^/L, respectively. Other laboratory findings were as follows: C-reactive protein (CRP), 2.69 mg/dL (normal range: <0.5 mg/dL); erythrocyte sediment rate (ESR), 27 mm/h (normal range: 0–20 mm/h); venous blood lactate, 2.2 mmol/L; procalcitonin, 0.145 ng/mL; C3 level, 136.8 mg/dL (normal range: 83–177 mg/dL); C4 level, 66.9 mg/dL (normal range: 15–45 mg/dL); CH50: 44.4 U/mL (normal range: 42–95 U/mL); total bilirubin, 0.94 mg/dL; direct Coombs test, positive; d-dimer, 4.16 µg/mL FEU; fibrinogen, 338 mg/dL; anti-thrombin-III, 104%; ferritin, 2249.20 ng/mL; and soluble interleukin-2 receptor, 2269 U/mL; antinuclear antibody, negative; blood and urine cytomegalovirus polymerase chain reaction (PCR), negative; Epstein–Barr virus VCA IgM and PCR, negative; parvovirus B19 PCR, negative; anti-HAV IgM, negative; anti-HCV, negative; HAV; HBs Ag, negative; HIV Ag/Ab, negative; tsutsugamushi Ab, negative; Hantaan virus Ab, negative; *Leptospira* Ab, negative; Widal test, O (<1:20), H (<1:20); and mycoplasma IgM, equivocal. Although her mycoplasma IgM was equivocal, she had no signs of infection or respiratory symptoms. Thus, she did not receive antibiotics.

Her autoimmune antibody panel (lupus anticoagulant, ANCA [MPO Ab], ANCA [PR3 Ab], anti-ds DNA antibody IgG, anti-ds DNA antibody IgM, Sm-Ab, anti-Smith antibody, SS-A/Ro Ab, anti-Ro antibody, SS-B/La Ab, anti-La antibody, anti-RNP antibody, anti-cardiolipin IgG, cardiolipin antibody IgG, anti-cardiolipin IgM, cardiolipin antibody IgM, anti-phospholipid IgG, anti-phospholipid IgM, anti-B2-GP1 IgG Ab, anti-B2-GP1 IgM Ab, and anti-ds-DNA IgM) and HLA-B27 DNA were negative.

**Figure 2:**
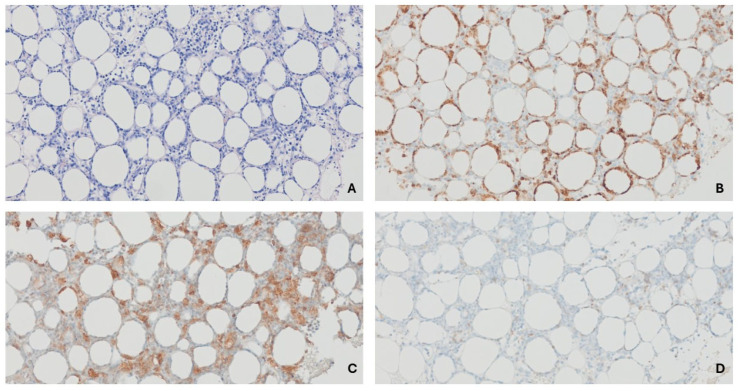
(**A**) Atypical lymphocytes infiltrate the subcutaneous fat lobules, displaying a rimming pattern around adipocytes with associated karyorrhectic debris (H&E, ×100). (**B**) Atypical cells are positive for CD8, highlighting the rimming of adipocytes (CD8, ×100). (**C**) Some CD4-positive cells are present, most of which are macrophages. The cells rimming the adipocytes are negative for CD4 (CD4 ×100). (**D**) Immunohistochemical staining for TCRδ is negative (TCRδ, ×100).

On the second day of hospitalization, a punch biopsy was performed on the subcutaneous skin of the patient’s left arm. The results showed lobular panniculitis with atypical lymphocytic infiltration, consistent with SPTCL (Figure 2). Subsequently, BIOMED-2 clonality assays detected positive clonal T-cell receptor (TCR)-γ chain gene rearrangement. However, the Epstein–Barr encoding region in situ hybridization test was negative.

**Figure 3:**
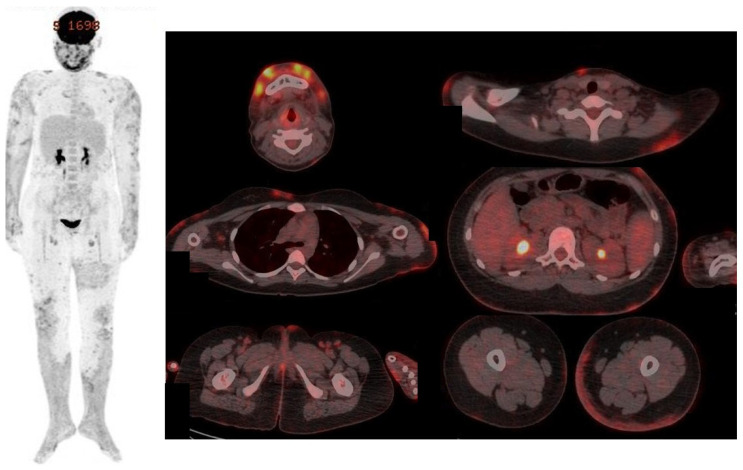
PET-CT image showing subcutaneous soft-tissue strandings and multifocal infiltrations and diffuse and heterogenous FDG uptake throughout the upper and lower extremities, face, trunk, and vulva.

Positron emission tomography–computed tomography (PET-CT) revealed subcutaneous soft-tissue strandings and multifocal infiltrations and diffuse and heterogenous fluorodeoxyglucose (FDG) uptake throughout the upper and lower extremities, face, right anterior chest wall, abdomen, lower back, and vulva (Figure 3). Several FDG-avid and/or enlarged lymph nodes were noted in the axillae and inguinal regions.

The patient was administered first-generation cephalosporin for 2 days. This was then switched to third-generation cephalosporin, and doxycycline was added. NSAIDs, including naproxen and acetaminophen, were also administered, but her fever persisted. On the 7th day of hospitalization, methylprednisolone (20 mg) was intravenously administered thrice daily in combination with cyclosporin after reviewing the biopsy results. Her fever subsided the following day.

On the 10th day of hospitalization, intravenous methylprednisolone was changed to oral prednisolone (20 mg) thrice a day. On the 13th day, blood test results were as follows: ferritin, 856.95 ng/mL; white blood cell count, 10.95 K/µL; CRP, 0.12 mg/dL; and ESR, 4 mm/H, indicating improvement in inflammatory signs. (Table 1) Next-generation sequencing identified a heterozygous C9 gene mutation (c.346C>T, p.Arg116Ter).

The patient was discharged on the 15th day of hospitalization. After discharge, prednisolone was gradually reduced over 3 months and discontinued, and cyclosporine was discontinued 1 month after prednisolone discontinuation. The patient has remained symptom-free for over a year.

## 3. Discussion

SPTCL is a rare cytotoxic T-cell lymphoma accounting for <1% of all peripheral T-cell lymphomas. The WHO lymphoma classification (2016) and the WHO–European Lymphoma Study and Treatment Group classification (2018) define SPTCL as tumors expressing α/β TCR gene recombination. Tumors expressing γ/δ TCR are classified separately as primary cutaneous γ/δ T-cell lymphoma.

SPTCL has nearly equal incidence between males and females and can occur in children and adults. According to a large case series, the median age at presentation for the two subtypes ranges from 36 years to 59 years [16].

SPTCL is a neoplasm of cytotoxic CD8+ T α/β cells with tropism for subcutaneous adipose tissue [17]. HLH has been mainly associated with poor outcomes.

Because SPTCL has overlapping clinicopathologic features with nonneoplastic panniculitides and other T-cell lymphomas involving the skin, SPTCL diagnosis is relatively difficult and based on the patient’s histopathologic features, immunophenotype, and clinical manifestations. Accordingly, SPTCL must be differentiated from primary cutaneous γ/δ T-cell lymphoma, extranodal natural killer/T-cell lymphoma, and lupus panniculitis. Lupus panniculitis is a group of diseases characterized by subcutaneous fat inflammation. Its clinical manifestations are similar to those of SPTCL. However, histopathologic examination reveals T cells, B cells, and plasma cells with fibrinoid changes in the connective tissue surrounding the blood vessels and lacks cytologic atypia. CD4+ and CD8+ T cells are present along with a good number of plasmacytoid dendritic cells (CD123+). Moreover, clonal TCR gene rearrangement is absent.

Because of the rare occurrence of such NHL subtypes during childhood, prospective therapeutic trials are lacking. Currently, most documented instances of SPTCL in pediatric populations are singular case reports [18]. In a study from China, the median age of 18 pediatric patients was 11.1 years, and 3 patients were less than 1 year of age [2].

Approximately 20% of pediatric patients with SPTCL have HLH, which is associated with an aggressive clinical course and poor OS rate. In one study, 7 out of 18 (38.9%) pediatric patients with HLH were diagnosed with suspected HLH and primarily treated with immunomodulatory agents [2]. However, the presence of HLH does not necessarily lead to a poor prognosis as remission can be achieved through high-dose chemotherapy [8].

Adult patients with SPTCL often exhibit unsatisfactory response rates to traditional anthracycline-based combination chemotherapy [19], making immunotherapy an important alternative. By contrast, pediatric patients demonstrate a relatively superior response to chemotherapy, with reports suggesting the potential for sustained disease control and high survival rates. In fact, one study reported a remarkable treatment effect in an infant who received five cycles of chemotherapy, with a 90% reduction in tumor volume [18]. This suggests differences in tumor biological characteristics or immune environments between pediatric and adult patients.

SPTCL treatment lacks a standardized therapeutic protocol. However, it encompasses immunosuppressive therapies (e.g., steroids with or without low-dose methotrexate, CyA, or hydroxychloroquine) and may involve chemotherapy regimens (e.g., CHOP). In a study comparing chemotherapy with CyA-based immunosuppressive therapy, a Thai lymphoma study group concluded that regimens incorporating cyclosporine yielded a superior CR rate relative to chemotherapy in patients diagnosed with SPTCL [9]. Patients administered a cyclosporine-based regimen exhibited a significantly elevated CR rate compared to those receiving chemotherapy (87% vs. 58.3%). Cyclosporine treatment decreased the incidence of progressive disease compared to chemotherapy (4.4% vs. 25%), resulting in a 5-year survival rate of 92%. The 5-year OS rates were 98% and 87% for the cyclosporine and chemotherapy cohorts, respectively. Furthermore, the 5-year progression-free survival rate was 72.4% for the cyclosporine cohort. The study concluded that CSA-based regimens are the preferred initial treatment for newly diagnosed cases of SPTCL, particularly in patients with limited skin involvement.

However, not all findings are consistent. Two studies conducted in France showed conflicting results. In one study involving 27 patients, the CR rate for immunosuppressive therapy was 81.2%, which was significantly higher than that for chemotherapy (28.5%) [20]. However, in another large retrospective study involving 70 patients, no significant difference in CR rates was observed between the two treatment groups [14]. This heterogeneity highlights the challenges in drawing standardized conclusions because of SPTCL’s rarity and the diversity of patient populations.

HLH is present in 15–20% of patients with SPTCL and is one of the most important factors determining prognosis [21]. The incidence of HLH is particularly high in children and young patients, indicating the necessity of aggressive treatment. In HLH cases, the 5-year OS rate can decrease by nearly half, making prompt diagnosis and aggressive intervention essential.

Recent studies have revealed a high frequency of HAVCR2 gene mutations in pediatric and young patients with SPTCL. According to a systematic review, the average age of onset in the HAVCR2 mutation group was 14.55 years, which was significantly lower than that in the non-mutation group (25.16 years). Moreover, the autoantibody positivity rate was very high (71.4%) [6]. This suggests that the disease may manifest in a form similar to autoimmune diseases in pediatric patients with HAVCR2 mutations, emphasizing the importance of genetic testing and differentiating from autoimmune diseases during diagnosis.

The C9 gene encodes complement component 9, a critical part of the membrane attack complex (MAC) in the complement system, which helps eliminate pathogens and damaged cells [22]. Heterozygous C9 gene mutations, including the c.346C>T (p.Arg116Ter) mutation in our patient, can lead to partial dysfunction of the complement system. Although homozygous C9 gene mutations are more strongly associated with significant clinical manifestations, heterozygous mutations may cause milder or variable symptoms, often depending on environmental or immunological stressors. Heterozygous C9 gene mutations may impair MAC formation, reducing the ability to lyse certain bacteria, particularly encapsulated organisms such as *Neisseria meningitidis* [22,23,24]. Moreover, partial complement dysfunction can disrupt immune regulation, potentially contributing to autoimmune conditions [25,26]. Heterozygous C9 gene mutations have been linked to an increased risk of systemic lupus erythematosus or lupus-like syndromes, characterized by joint pain, skin rashes (e.g., malar rash), photosensitivity, and fatigue. The heterozygous C9 gene mutation (c.346C>T, p.Arg116Ter) likely results in normal or mildly reduced CH50 levels. However, specific complement function testing was not performed in this case. Furthermore, the partial complement dysfunction and disruption of immune regulation caused by the C9 gene mutation are inferential hypotheses, so further research is needed.

## 4. Conclusions

In summary, we report a case of adolescent SPTCL treated with immunosuppressive therapy and suggest that immunosuppressive therapy should be considered before chemotherapy in pediatric patients with SPTCL but without HLH.

## Figures and Tables

**Table 1:** Laboratory findings of the patient.

Days After Admission	1	7	13	22	36	50
ESR (mm/H)	27	8	4	2	2	4
Ferritin (ng/mL)	3612.31	2249.2	856.95	307.2	101.38	45.09
WBC (K/μL)	4.58	2.68	10.95	16.1	12.06	10.41
LDH (IU/L)	636	687	349	245	208	204
CRP (mg/dL)	2.69	6.2	0.12	1.06	0.03	0.04
D-dimer (μg/mL FEU)	4.16	1.74	1.25	1.06	0.36	0.26

## Data Availability

The data presented in this study are available on request from the corresponding author.
